# Admission high-sensitivity cardiac troponin T and clinical outcomes after acute ischemic stroke: a retrospective cohort study with exploratory nonlinear analysis

**DOI:** 10.3389/fneur.2026.1882492

**Published:** 2026-10-22

**Authors:** Yisheng Chen, Suhua Liu, Hua Xue, Rui Wang, Rongfang Wen, Zhaoxiang Xiao, Xuerong Sheng, Peifeng Chen, Na Song, Yongkun Li

**Affiliations:** 1 Shengli Clinical Medical College of Fujian Medical University, Fuzhou, Fujian, China; 2 Department of Neurology, Shanghang County Hospital, Longyan, Fujian, China; 3 Department of Neurology, Fuzhou University Affiliated Provincial Hospital, Fuzhou, Fujian, China

**Keywords:** acute ischemic stroke, high-sensitivity cardiac troponin T (hs-cTnT), modified Rankin scale (mRS), mortality, nonlinear association, restricted cubic spline (RCS)

## Abstract

**Background:**

Elevated high-sensitivity cardiac troponin T (hs-cTnT) is frequently observed in patients with acute ischemic stroke (AIS) and has been associated with adverse outcomes. We aimed to evaluate the association of admission hs-cTnT with 90-day functional outcome and all-cause mortality during follow-up, with particular attention to potential nonlinear risk patterns across the biomarker range.

**Methods:**

This single-center retrospective cohort study included 564 patients with AIS admitted between January 2022 and October 2024. The primary outcomes were modified Rankin Scale (mRS) at 90 days and long-term all-cause mortality. Multivariable logistic regression was used for poor 90-day functional outcome and Cox proportional hazards models were used for all-cause mortality during follow-up. Restricted cubic spline (RCS) analyses were performed to explore nonlinear dose–response relationships, and segmented regression models were used to estimate exploratory turning points.

**Results:**

Among 564 patients, 212 (37.6%) had elevated hs-cTnT (>14 ng/L). Elevated hs-cTnT was independently associated with poor 90-day mRS (adjusted OR 1.93, 95% CI, 1.22–3.05; *p* = 0.005) and all-cause mortality during follow-up (fully adjusted HR 4.12, 95% CI, 2.31–7.48; *p* < 0.001). During a median follow-up of 17 months (IQR 12–26 months), Kaplan–Meier analysis demonstrated significantly lower overall survival in patients with elevated hs-cTnT (*p* < 0.001). RCS analysis revealed significant nonlinear associations between hs-cTnT and both endpoints (*p* < 0.001). Exploratory model-derived turning points were observed at 28.42 ng/L for functional outcome and 31.68 ng/L for mortality; these should be interpreted as exploratory rather than validated clinical cutoffs. Below these values, risk increased more steeply; above them, no statistically significant further increase in risk was observed.

**Conclusion:**

Admission hs-cTnT was independently associated with 90-day functional outcome and all-cause mortality during follow-up in patients with AIS. The association appeared to be nonlinear, with model-derived turning points at approximately 28–32 ng/L that should be interpreted as exploratory rather than definitive clinical cutoffs. Clinically, hs-cTnT may be useful as a complementary marker for identifying patients at higher risk who may warrant closer neuro-cardiac evaluation, although its incremental predictive value has not been formally assessed.

## Highlights

- Elevated hs-cTnT measured within 24 h of admission (>14 ng/L) was independently associated with poor 90-day functional outcome and increased all-cause mortality in AIS.
- The hs-cTnT–outcome association was significantly nonlinear (both *p* < 0 0.001).
- Exploratory segmented regression identified model-derived turning points at approximately 28–32 ng/L.
- hs-cTnT may serve as an adjunctive marker of prognosis rather than as a standalone treatment threshold.
- Prospective multicenter studies are warranted to validate these exploratory turning points and their clinical utility.

## 1 Introduction

Acute ischemic stroke (AIS) is a leading cause of mortality and disability worldwide. Its high incidence, disability, and mortality rates impose a substantial burden on patients’ families and on healthcare systems. According to the latest Global Burden of Disease (GBD) 2021 analysis, stroke remains the second leading cause of death and the third leading cause of disability globally, and ischemic stroke accounts for approximately 62% of all stroke cases (1, 2). Despite significant advances in therapeutic strategies for AIS in recent years, including the widespread use of intravenous thrombolysis and endovascular thrombectomy (3, 4), a considerable proportion of patients continue to experience persistent functional impairment. Identifying patients at high risk of a poor outcome during the acute phase is therefore clinically important, both for individualizing management and for informing discussions about prognosis; observational associations, however, cannot by themselves establish treatment recommendations.

Cardiac troponin (cTn) is an essential component of the contractile regulatory protein complex in cardiomyocytes, comprising troponin C (cTnC), troponin I (cTnI), and troponin T (cTnT) (5). When the integrity of the cardiomyocyte membrane is compromised, troponin is released into the circulation; elevated serum levels therefore serve as a highly specific marker of myocardial injury (5). Conventional assays have limited sensitivity and may fail to detect minor myocardial injury. With advances in laboratory technology, high-sensitivity cardiac troponin T (hs-cTnT) assays have emerged and are now used widely in acute cardiac care (6). In addition to their role in acute coronary syndrome, hs-cTnT assays also have prognostic value in several non-cardiac conditions (7).

In recent years, numerous studies have examined the association between hs-cTnT and prognosis after ischemic stroke (8–11). Elevated cTnT levels on admission are observed in 30–60% of patients, even in the absence of acute coronary syndrome or myocardial infarction (8, 10). The mechanisms underlying hs-cTnT elevation in stroke are likely multifactorial and involve brain-heart interactions, autonomic dysfunction, and systemic inflammatory activation (11). Most studies of cardiac troponin in acute ischaemic stroke have analysed the biomarker dichotomously against the assay-specific 99th-percentile upper reference limit, and an updated systematic review and meta-analysis in this population likewise pooled dichotomised exposures, so that the shape of the association could not be characterised (12). In general and cardiovascular populations, by contrast, continuous modelling has shown that the relationship between high-sensitivity cardiac troponin and cardiovascular events is graded and not log-linear across the measurable range (13, 14). In patients with ischaemic stroke or transient ischaemic attack, hs-cTnT carries risk information within the range conventionally regarded as normal (15), which implies that a single dichotomy at 14 ng/L discards information. To our knowledge, however, the dose–response shape of hs-cTnT measured within 24 h of admission against 90-day functional outcome and all-cause mortality after acute ischaemic stroke has not been described. We therefore analysed hs-cTnT as a continuous variable using restricted cubic splines, and used segmented regression only as an exploratory description of where the gradient changed.

This study aimed to evaluate whether hs-cTnT measured within 24 h of admission was independently associated with 90-day functional outcome and all-cause mortality during follow-up following acute ischemic stroke, and whether these associations were nonlinear across the biomarker range. Rather than relying solely on a binary definition of troponin elevation, we analyzed hs-cTnT as a continuous variable and used restricted cubic spline methods to explore potential dose–response patterns and model-derived turning points. By clarifying the shape of this association, we sought to determine whether hs-cTnT may have clinical value as an adjunctive marker of prognosis and for prompting broader neuro-cardiac assessment, rather than as a standalone threshold for individualized treatment decisions.

## 2 Methods

### 2.1 Study design and population

This was a single-center retrospective cohort study designed to investigate the association of hs-cTnT measured within 24 h of admission with 90-day functional outcome and all-cause mortality during follow-up in patients with acute ischemic stroke, with additional exploratory analyses of nonlinear patterns and model-derived turning points. The study protocol was approved by the Ethics Committee of Shanghang County Hospital (approval number: 20251202). Retrospective ethics approval was granted on December 2, 2025. Given the retrospective nature of the study, which involved only the collection and analysis of medical records without any additional intervention, the requirement for informed consent was waived by the Ethics Committee. This study was conducted in accordance with the Declaration of Helsinki and adhered to the Strengthening the Reporting of Observational Studies in Epidemiology (STROBE) guidelines (16).

Clinical data from patients hospitalized for acute ischemic stroke in the Department of Neurology at Shanghang County Hospital between January 2022 and October 2024 were retrospectively collected. Eligible patients were screened through the hospital electronic medical record system. Inclusion criteria were as follows: (1) age ≥18 years; (2) diagnosis of AIS according to the Chinese Guidelines for the Diagnosis and Treatment of Acute Ischemic Stroke and confirmed by cranial computed tomography (CT) or magnetic resonance imaging (MRI); (3) time from symptom onset to admission ≤72 h; (4) completion of hs-cTnT testing within 24 h of admission; and (5) availability of the variables required for the prespecified analyses, namely hs-cTnT measured within 24 h of admission, 90-day functional status or ascertainable vital status, and the baseline covariates listed in Section 2.2. Exclusion criteria included: (1) hemorrhagic stroke or subarachnoid hemorrhage at admission; (2) documented myocardial infarction or acute coronary syndrome at admission; (3) severe cardiac diseases, including acute or chronic heart failure, severe valvular heart disease, hypertrophic cardiomyopathy, or dilated cardiomyopathy; (4) severe hepatic or renal insufficiency (Child-Pugh class C for liver function or estimated glomerular filtration rate [eGFR] < 30 mL/min/1.73 m^2^); (5) malignancy, severe infection, or autoimmune disease; (6) major surgery or severe trauma within 30 days before admission; (7) recent cardiac intervention (e.g., percutaneous coronary intervention or cardiac surgery) within 4 weeks before admission; (8) pregnancy or lactation; and (9) significant missing data precluding effective analysis. Among 1,447 patients initially screened, 883 were excluded according to the predefined eligibility criteria, and 564 patients were ultimately included in the final analysis. The detailed reasons for exclusion are shown in Figure 1.

**Figure 1:**
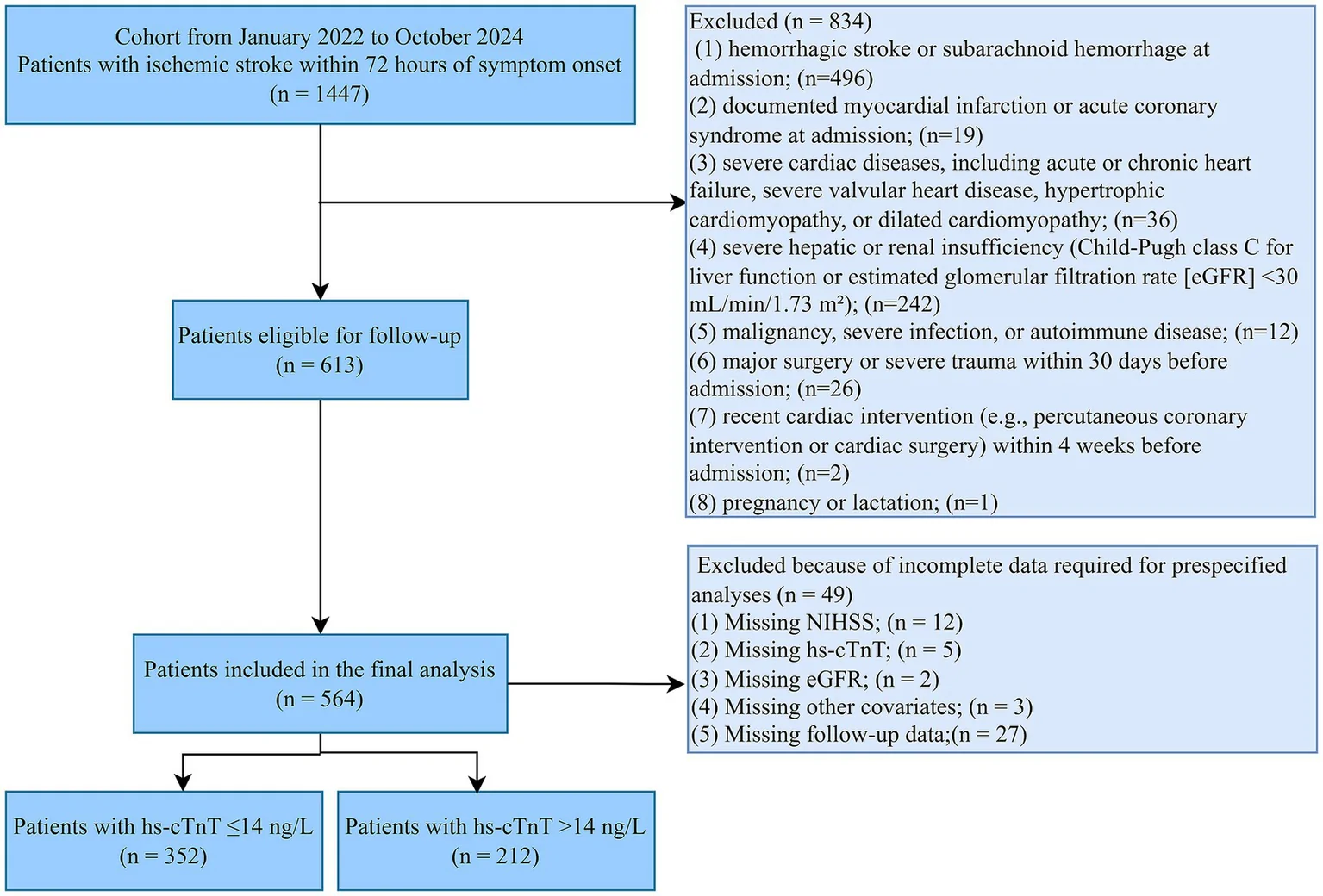
Flowchart of patient selection. Patients were screened from January 2022 to October 2024. Exclusions were categorized into clinical eligibility criteria and incomplete data required for prespecified analyses. eGFR indicates estimated glomerular filtration rate; ACS, acute coronary syndrome; hs-cTnT, high-sensitivity cardiac troponin T.

### 2.2 Data collection

Clinical data were retrospectively collected from the hospital electronic medical record system by investigators who had undergone standardized training and used a standardized data extraction form. The collected data included: (1) demographic characteristics, including age, and sex; (2) medical history, including hypertension, diabetes mellitus, atrial fibrillation, coronary artery disease, hyperlipidemia, history of previous stroke or transient ischemic attack (TIA), smoking history, and alcohol consumption history; (3) clinical characteristics on admission, including systolic and diastolic blood pressure and National Institutes of Health Stroke Scale (NIHSS) score; (4) laboratory indicators, including hs-cTnT levels within 24 h of admission; and (5) imaging findings from cranial CT or MRI.

Venous blood samples (3 mL) were collected as part of routine clinical care within 24 h of admission. Samples were placed in vacuum tubes containing separation gel, allowed to stand at room temperature for 30 min, and centrifuged at 3000 rpm for 10 min. Serum was separated and tested within 2 h. hs-cTnT levels were measured using the Roche Elecsys Troponin T hs assay on a Cobas e601 automated electrochemiluminescence immunoassay analyzer. The assay had a linear range of 3–10,000 ng/L and a lower detection limit of 3 ng/L; the lowest concentration with a coefficient of variation <10% was 13 ng/L. According to the manufacturer specifications and international guidelines, the 99th percentile upper reference limit (URL) for hs-cTnT was 14 ng/L (6, 17). In the primary analyses, hs-cTnT was analyzed as a continuous variable to preserve the full information of the biomarker and avoid potential information loss associated with arbitrary categorization. For the main categorical analysis, hs-cTnT was dichotomized at 14 ng/L based on the assay-specific 99th percentile URL. For sensitivity analyses, hs-cTnT was additionally categorized according to its empirical quartile distribution in the study population. To facilitate clinical interpretation, continuous hs-cTnT was also rescaled to a 10 ng/L increment, and the corresponding effect estimates were obtained using the same regression models and covariates.

### 2.3 Outcomes

The primary outcome measures of this study were as follows: (1) Functional outcome at 90 days: functional status at 90 days post-stroke was assessed using the modified Rankin Scale (mRS). Data on mRS scores were obtained from outpatient follow-up records, inpatient records, and telephone interviews (18). mRS scores range from 0 to 6, where 0 indicates no symptoms and 6 indicates death. Based on previous studies, an mRS score of 0–2 was defined as a good functional outcome and an mRS score of 3–6 as a poor functional outcome (19). (2) all-cause mortality during follow-up: all-cause mortality was recorded by reviewing hospital death records, conducting telephone follow-up, and querying the local population death registry system. For the mortality analysis, time zero was the date of hospital admission, the point at which exposure status (hs-cTnT within 24 h of admission) was ascertained; survival time was calculated from admission to death or to the last follow-up date (December 31, 2024), whichever came first. Because patients had to survive until blood sampling to enter the cohort, the interval between symptom onset and admission and between admission and sampling were not counted as time at risk. To examine whether the choice of time origin affected the results, all Cox models were refitted with stroke onset as the origin, both with delayed entry at admission (left truncation) and as a simple onset-based origin. Secondary outcome measures included recurrent cerebrovascular events (ischemic or hemorrhagic stroke).

### 2.4 Statistical analysis

Categorical variables were expressed as frequencies (percentages) and compared using the chi-square test or Fisher’s exact test, as appropriate. Continuous variables were presented as mean ± standard deviation (SD) for normally distributed data and as median (interquartile range [IQR]) for non-normally distributed data, including hs-cTnT levels and follow-up duration. Between-group comparisons were performed using the Student’s *t*-test or Mann–Whitney U test, respectively. The associations between hs-cTnT levels and clinical outcomes were evaluated using multivariable logistic regression for 90-day poor functional outcome (mRS ≥ 3). For long-term all-cause mortality, Cox proportional hazards regression models were used to estimate hazard ratios (HRs) and 95% confidence intervals (CIs). The proportional hazards assumption was assessed using Schoenfeld residual-based tests for the multivariable Cox regression model. A *p* value <0.05 was considered indicative of potential violation of the proportional hazards assumption. Cox proportional hazards models were used to assess the relationship between hs-cTnT levels and all-cause mortality during follow-up. Covariates were selected *a priori* based on established clinical knowledge and previously reported predictors of functional outcome and mortality after acute ischemic stroke, as well as factors known to influence hs-cTnT concentrations. We did not use data-driven variable selection methods such as stepwise regression, LASSO, or univariable screening. Age and sex were included because both are strong predictors of stroke outcome and are associated with baseline hs-cTnT levels. Smoking status, hypertension, diabetes mellitus, coronary heart disease, and prior cerebral infarction were included as established vascular risk factors that contribute to both stroke prognosis and myocardial injury. Baseline NIHSS score was included because stroke severity is the strongest determinant of post-stroke functional outcome and mortality. History of renal impairment and eGFR were included because renal function affects hs-cTnT clearance and is an independent prognostic factor after stroke. Three models were constructed. Model 1 was unadjusted. Model 2 was adjusted for age, sex, and smoking status. Model 3 was further adjusted for baseline NIHSS score, hypertension, diabetes mellitus, coronary heart disease, prior cerebral infarction, history of renal impairment, and eGFR. Baseline NIHSS score was entered as a continuous variable. Multicollinearity was assessed using variance inflation factors (VIF), and all variables had VIF values below 5. Multiplicative interactions were tested by including cross-product terms in the models and were evaluated using likelihood ratio tests. To explore potential nonlinear dose–response relationships, restricted cubic spline (RCS) functions were incorporated into the regression models (20). Four knots were placed at the 5th, 35th, 65th, and 95th percentiles of hs-cTnT distribution (3.00, 7.11, 14.6395, and 52.3105 ng/L, respectively), with 10.545 ng/L as the reference value. Four knots were selected to balance flexibility in modeling nonlinear associations and avoidance of unnecessary model complexity. Segmented (two-piecewise) regression analyses were further performed to estimate potential inflection points; these analyses were considered exploratory rather than prespecified clinically actionable thresholds (21). Kaplan–Meier survival curves, with time measured from admission, were constructed to evaluate all-cause mortality and recurrent cerebrovascular events, stratified by hs-cTnT levels (>14 ng/L vs. ≤14 ng/L). For recurrent cerebrovascular events, death was considered a competing event because patients who died could no longer experience subsequent recurrent events. Therefore, cumulative incidence functions were estimated, and Fine–Gray competing risk regression models were applied. Subdistribution hazard ratios (SHRs) with 95% confidence intervals (CIs) were calculated. The multivariable model was adjusted for age, sex, admission NIHSS, eGFR, and coronary heart disease. Differences between groups were assessed using the log-rank test. All statistical analyses were performed using R software (version 4.3.2; R Foundation for Statistical Computing, Vienna, Austria). A two-sided *p* value < 0.05 was considered statistically significant.

## 3 Results

### 3.1 Baseline characteristics

A total of 564 patients with AIS were included in the final analysis. Based on the 99th percentile upper reference limit (14 ng/L), patients were stratified into two groups: the normal hs-cTnT group (≤14 ng/L, *n* = 352) and the elevated hs-cTnT group (>14 ng/L, *n* = 212). The baseline characteristics of the study population are summarized in Table 1. Patients in the elevated hs-cTnT group were significantly older than those in the normal group (78.28 ± 9.78 years vs. 69.03 ± 11.29 years, *p* < 0.001). Regarding stroke severity, the distribution of NIHSS categories differed significantly between the two groups (*p* < 0.001), with a higher proportion of patients in the elevated hs-cTnT group presenting with moderate-to-severe neurological deficits. In terms of comorbidities, the prevalence of hypertension (81.60% vs. 73.30%, *p* = 0.024), coronary heart disease (19.81% vs. 9.38%, *p* < 0.001), and prior cerebral infarction (28.30% vs. 15.34%, *p* < 0.001) was significantly higher in the elevated hs-cTnT group. Laboratory findings revealed that patients with elevated hs-cTnT levels had significantly lower eGFR (65.49 ± 17.37 mL/min/1.73 m^2^ vs. 79.62 ± 17.80 mL/min/1.73 m^2^, *p* < 0.001). In addition, diastolic blood pressure on admission was lower in the elevated hs-cTnT group (84.48 ± 12.27 mmHg vs. 87.35 ± 13.18 mmHg, *p* = 0.010).

**Table 1:** Baseline characteristics of patients stratified by hs-cTnT levels.

Variables	Total (*n* = 564)	≤14 ng/L (*n* = 352)	>14 ng/L (*n* = 212)	*p* value
Age (years), mean ± SD	72.51 ± 11.64	69.03 ± 11.29	78.28 ± 9.78	<0.001
Age group, *n* (%)				<0.001
≤60	91 (16.13)	79 (22.44)	12 (5.66)	
>60	473 (83.87)	273 (77.56)	200 (94.34)	
Sex, *n* (%)				0.716
Male	338 (59.93)	213 (60.51)	125 (58.96)	
Female	226 (40.07)	139 (39.49)	87 (41.04)	
NIHSS categories, *n* (%)				<0.001
0–1	147 (26.25)	105 (30.00)	42 (20.00)	
2–4	240 (42.86)	155 (44.29)	85 (40.48)	
5–15	159 (28.39)	89 (25.43)	70 (33.33)	
16–20	9 (1.61)	1 (0.29)	8 (3.81)	
≥21	5 (0.89)	0 (0.00)	5 (2.38)	
SBP (mmHg), mean ± SD	151.25 ± 21.34	150.51 ± 21.37	152.48 ± 21.30	0.290
DBP (mmHg), mean ± SD	86.27 ± 12.91	87.35 ± 13.18	84.48 ± 12.27	0.010
hs-cTnT (ng/L), median (Q1–Q3)	10.54 (5.10, 17.09)	6.53 (3.30, 9.78)	20.41 (16.34, 30.85)	<0.001
eGFR (mL/min/1.73 m^2^), mean ± SD	74.31 ± 18.91	79.62 ± 17.80	65.49 ± 17.37	<0.001
Follow-up time (months), median (Q1–Q3)	17.00 (12.00, 26.00)	19.00 (12.00, 27.00)	14.00 (11.00, 25.25)	<0.001
Hypertension, *n* (%)				0.024
No	133 (23.58)	94 (26.70)	39 (18.40)	
Yes	431 (76.42)	258 (73.30)	173 (81.60)	
Diabetes mellitus, *n* (%)				0.940
No	424 (75.18)	265 (75.28)	159 (75.00)	
Yes	140 (24.82)	87 (24.72)	53 (25.00)	
Coronary heart disease, *n* (%)				<0.001
No	489 (86.70)	319 (90.62)	170 (80.19)	
Yes	75 (13.30)	33 (9.38)	42 (19.81)	
Prior cerebral infarction, *n* (%)				<0.001
No	450 (79.79)	298 (84.66)	152 (71.70)	
Yes	114 (20.21)	54 (15.34)	60 (28.30)	
History of renal impairment, *n* (%)				0.438
No	555 (98.40)	348 (98.86)	207 (97.64)	
Yes	9 (1.60)	4 (1.14)	5 (2.36)	
Smoking, *n* (%)				0.071
No	409 (72.52)	246 (69.89)	163 (76.89)	
Yes	155 (27.48)	106 (30.11)	49 (23.11)	
Infarct territory, *n* (%)				0.011
Anterior circulation	357 (63.30)	227 (64.49)	130 (61.32)	
Posterior circulation	126 (22.34)	86 (24.43)	40 (18.87)	
Multiple territories	81 (14.36)	39 (11.08)	42 (19.81)	
Onset to admission ≤72 hours	8.00 (5.00, 11.12)	8.00 (4.50, 12.00)	7.50 (5.00, 11.00)	0.363
Admission to sampling (Min)	9.00 (8.00, 12.00)	9.00 (8.00, 12.00)	9.00 (8.00, 11.25)	0.161

Continuous variables are presented as mean ± standard deviation (SD) for normally distributed data and as median (interquartile range [IQR]) for non-normally distributed data, including hs-cTnT and follow-up duration. Categorical variables are expressed as number (percentage). Comparisons between groups were performed using the Student’s *t*-test or Mann–Whitney U test for continuous variables and the chi-square test or Fisher’s exact test for categorical variables, as appropriate. NIHSS score data were missing in four patients (2 in the hs-cTnT ≤14 ng/L group and 2 in the hs-cTnT >14 ng/L group), and percentages were calculated based on available data.

There were no statistically significant differences between the two groups in terms of sex, prevalence of diabetes mellitus, smoking history, systolic blood pressure, or time from onset to admission (all *p* > 0.05). Notably, the distribution of infarct territories differed significantly between the groups (*p* = 0.011), with a higher proportion of patients having infarcts involving multiple territories in the elevated hs-cTnT group (19.81% vs. 11.08%). Furthermore, patients in the elevated hs-cTnT group had significantly worse functional outcomes at 90 days (*p* < 0.001). The distribution of 90-day mRS scores according to hs-cTnT levels is illustrated in Figure 2, showing a clear shift toward worse functional status in patients with elevated hs-cTnT.

**Figure 2:**
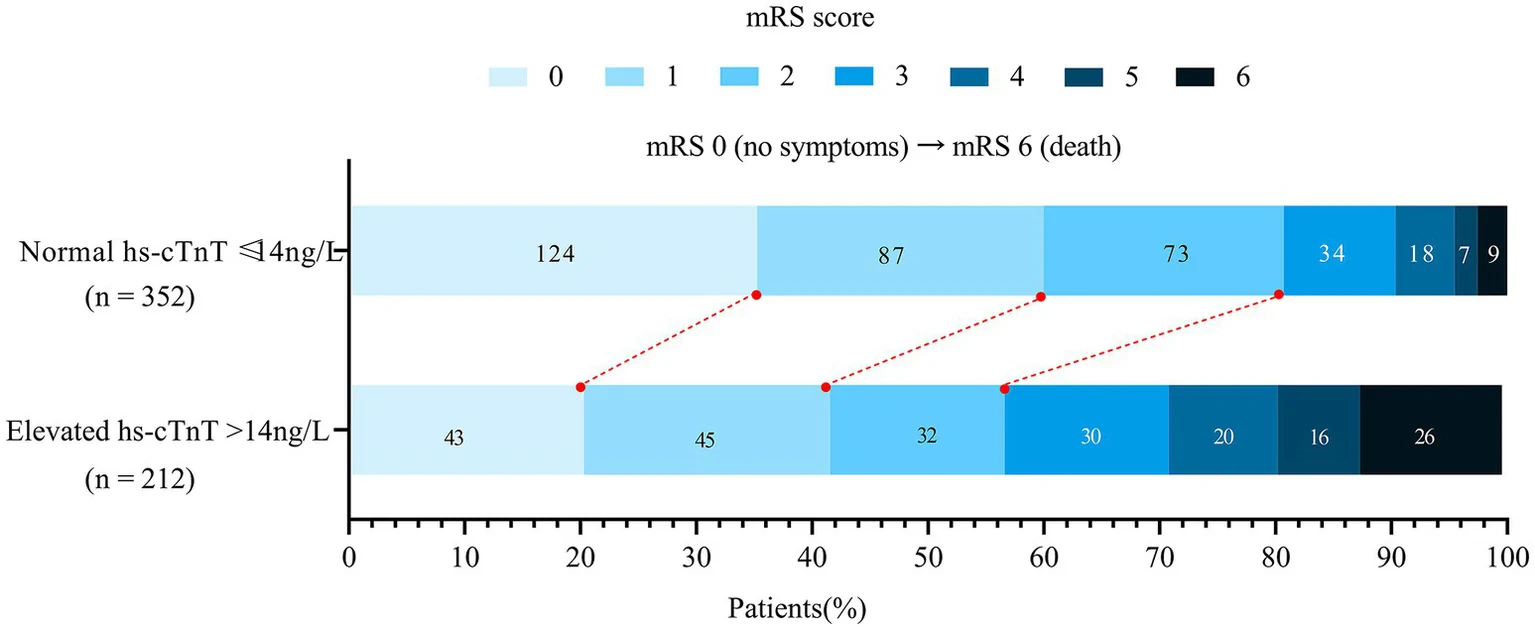
Distribution of modified Rankin Scale (mRS) scores at 90 days, stratified by admission high-sensitivity cardiac troponin T (hs-cTnT) level. The upper bar represents patients with normal hs-cTnT (≤14 ng/L, *n* = 352) and the lower bar represents patients with elevated hs-cTnT (>14 ng/L, *n* = 212). Each segment is colour-coded by mRS score from 0 (no symptoms, lightest blue) to 6 (death, darkest blue); the number inside each segment denotes the patient count, and segment width reflects the within-group percentage. Numbers inside segments are patient counts; segment widths are row percentages. Red dashed lines connect the cumulative proportions at mRS 0, mRS 0–1, and mRS 0–2 between the two groups, illustrating that a greater proportion of patients with lower hs-cTnT achieved a favourable outcome (mRS 0–2: 80.7% vs. 56.6%).

### 3.2 Association between hs-cTnT and clinical outcomes

To investigate the independent association between hs-cTnT and clinical outcomes, multivariable logistic regression analyses were performed for 90-day poor functional outcome, while Cox proportional hazards models were used for all-cause mortality during follow-up. The results are summarized in Table 2. Subgroup analyses are shown in Supplementary Figure S1.

**Table 2:** Multivariable analyses of hs-cTnT with 90-day functional outcome (logistic) and long-term all-cause mortality (Cox).

Outcome	hs-cTnT (ng/L)	Model 1 OR / HR (95% CI)	*p* value	Model 2 OR / HR (95% CI)	*p* value	Model 3 OR / HR (95% CI)	*p* value
90-day poor functional outcome (mRS 3–6)	>14 ng/L vs. ≤ 14 ng/L	3.20 (2.19–4.68)	<0.001	2.83 (1.91–4.18)	<0.001	1.93 (1.22–3.05)	0.005
Long-term all-cause mortality	>14 ng/L vs. ≤ 14 ng/L	6.24 (3.68–10.57)	<0.001	5.88 (3.44–10.05)	<0.001	4.12 (2.31–7.48)	<0.001

OR, odds ratio; HR, hazard ratio; CI, confidence interval; hs-cTnT, high-sensitivity cardiac troponin T; mRS, modified Rankin Scale; NIHSS, National Institutes of Health Stroke Scale; eGFR, estimated glomerular filtration rate. Model 1 was unadjusted. Model 2 was adjusted for age, sex, and smoking status. Model 3 was further adjusted for baseline NIHSS score, hypertension, diabetes mellitus, coronary heart disease, prior cerebral infarction, history of renal impairment, and eGFR. For 90-day poor functional outcome, estimates are ORs from logistic regression; for long-term all-cause mortality, estimates are HRs from Cox proportional hazards models. hs-cTnT was modeled as a binary variable (>14 ng/L vs ≤14 ng/L). Models 1 and 2 were fitted on 564 patients; Model 3 was fitted on the 560 patients with complete covariate data. Quartile and continuous analyses are presented in Supplementary Tables S1, S2.

For 90-day poor functional outcome, in the unadjusted Model 1, patients with hs-cTnT levels >14 ng/L had a 3.20-fold higher risk of poor functional outcome (mRS 3–6) than those with hs-cTnT levels ≤14 ng/L (unadjusted OR 3.20, 95% CI, 2.19–4.68; *p* < 0.001). After sequential adjustment for covariates (Models 2 and 3), the association remained statistically significant, with the OR decreasing to 2.83 (95% CI, 1.91–4.18; *p* < 0.001) in Model 2 and 1.93 (95% CI, 1.22–3.05; *p* = 0.005) in Model 3, indicating that elevated hs-cTnT was independently associated with an increased risk of poor functional outcome.

For all-cause mortality during follow-up, Cox proportional hazards models demonstrated that elevated hs-cTnT was consistently associated with increased mortality risk (Table 2). Compared with patients with hs-cTnT ≤14 ng/L, those with hs-cTnT >14 ng/L had a significantly higher hazard of death in Model 1 (unadjusted HR 6.24, 95% CI, 3.68–10.57; *p* < 0.001), Model 2 (adjusted HR 5.88, 95% CI, 3.44–10.05; *p* < 0.001), and Model 3 (adjusted HR 4.12, 95% CI, 2.31–7.48; *p* < 0.001). Quartile analysis showed a graded association: compared with the lowest quartile (<5.10 ng/L), the second and third quartiles had higher mortality risks, and the highest quartile (>17.09 ng/L) had a markedly increased risk (adjusted HR 10.66, 95% CI, 3.12–36.50; *p* < 0.001) (Supplementary Table S1). Each 10 ng/L increase in hs-cTnT was associated with higher odds of 90-day poor functional outcome (adjusted OR, 1.05; 95% CI, 1.01–1.10; *p* = 0.028) and higher risk of long-term all-cause mortality (adjusted HR, 1.007; 95% CI, 1.002–1.011; *p* = 0.004) in the fully adjusted models (Supplementary Table S2). Given the relatively limited number of mortality events, a parsimonious Cox model including hs-cTnT, age, sex, admission NIHSS, and eGFR was additionally performed as a sensitivity analysis. The association between elevated hs-cTnT (>14 ng/L) and long-term all-cause mortality remained significant in the parsimonious model (HR, 3.59; 95% CI, 1.98–6.50; *p* < 0.001), with an effect estimate directionally consistent with that of the fully adjusted model (HR, 4.12; 95% CI, 2.31–7.48; *p* < 0.001; Supplementary Table S3). The proportional hazards assumption was assessed using Schoenfeld residual-based tests. No statistically significant evidence of violation was observed for hs-cTnT (*p* = 0.081) or for any other covariate, and the global test was also non-significant (*p* = 0.361; Supplementary Table S4).

### 3.3 Kaplan–Meier survival analysis

During the median 17-month follow-up period, 149 outcome events were recorded, including 71 recurrent strokes (12.6%) and 78 all-cause deaths (13.8%). Recurrent strokes included 9 hemorrhagic strokes (1.6%) and 62 ischemic strokes (11.0%). In total, 11 patients experienced both recurrent stroke and all-cause death during the observation period. The cohort contributed a total person-time of 893.8 person-years, corresponding to an incidence rate of 8.7 per 100 person-years for all-cause mortality and 7.9 per 100 person-years for recurrent stroke.

Kaplan–Meier survival curves demonstrated a significant difference in the cumulative incidence of all-cause mortality between patients with elevated hs-cTnT (>14 ng/L) and those with lower levels (≤14 ng/L). During a median follow-up of 17 months, patients in the elevated hs-cTnT group showed markedly lower overall survival (log-rank *p* < 0.001), with an unadjusted HR of 6.24 (95% CI, 3.68–10.57; Model 1, Table 2) (Figure 3A). No patient died before hs-cTnT sampling, and no patient deaths occurred within 7 days of admission. The HR were essentially unchanged when stroke onset rather than admission was used as the time origin, with or without delayed entry at admission (fully adjusted HR 4.17 and 4.41, respectively, vs. 4.12 with admission as the origin; Supplementary Table S5). For recurrent cerebrovascular events, death was considered a competing event because patients who died could no longer experience subsequent recurrent events. Therefore, cumulative incidence functions were estimated and compared using Gray’s test, and Fine–Gray competing risk regression models were applied. The cumulative incidence curves of the two groups overlapped (Gray’s test *p* = 0.695), and after accounting for the competing risk of death no significant difference in recurrent cerebrovascular events was observed between patients with elevated and normal hs-cTnT levels. In the unadjusted analysis, elevated hs-cTnT was not associated with recurrent cerebrovascular events (subdistribution hazard ratio [SHR], 0.87; 95% CI, 0.50–1.51; *p* = 0.618). Similarly, after adjustment for age, sex, admission NIHSS, eGFR, and coronary heart disease, elevated hs-cTnT remained not significantly associated with recurrent cerebrovascular events (SHR, 1.05; 95% CI, 0.56–1.96; *p* = 0.877) (Supplementary Table S6). The cumulative incidence curves are shown in Figure 3B. These findings suggested that while elevated hs-cTnT was strongly associated with increased all-cause mortality, its association with recurrent cerebrovascular events was not evident after considering death as a competing event.

**Figure 3:**
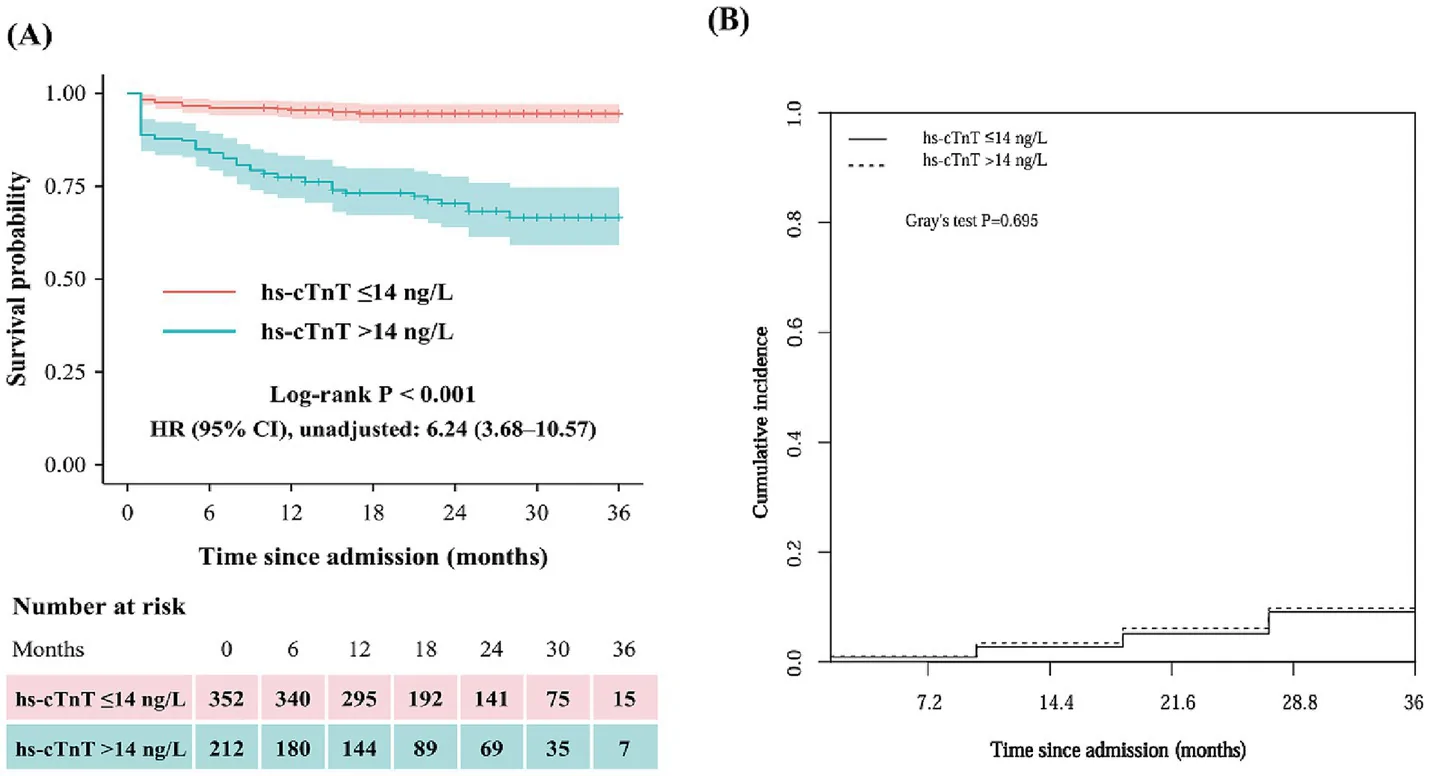
Survival and cumulative incidence according to hs-cTnT level. **(A)** Kaplan–Meier curves for overall survival (all-cause mortality); patients with hs-cTnT >14 ng/L had significantly lower survival than those with hs-cTnT ≤14 ng/L (log-rank *p* < 0.001; unadjusted Cox hazard ratio 6.24, 95% CI 3.68–10.57). **(B)** Cumulative incidence functions for recurrent cerebrovascular events with death treated as a competing event; the two groups did not differ (Gray’s test *p* = 0.695; unadjusted SHR 0.87, 95% CI 0.50–1.51, *p* = 0.618). Numbers at risk are shown separately for each panel and are computed for the endpoint displayed in that panel.

### 3.4 Nonlinear associations and turning points of hs-cTnT

Restricted cubic spline analysis demonstrated significant nonlinear associations between hs-cTnT and both primary endpoints (*p* < 0.001) (Figure 4). For 90-day functional outcome, segmented regression identified an estimated turning point at 28.42 ng/L. below this level, each 1 ng/L rise in hs-cTnT was associated with a 4.8% increase in the odds of a poor outcome (adjusted OR 1.048, 95% CI, 1.005–1.093; *p* < 0.001), whereas no statistically significant further increase in risk was observed above this value (*p* = 0.328). A similar pattern was observed for mortality, with an estimated turning point of 31.68 ng/L. below this level, mortality risk increased by 9.6% per 1 ng/L increase in hs-cTnT (HR 1.096, 95% CI, 1.045–1.149; *p* < 0.001), whereas no statistically significant further increase in risk was observed above the turning point (*p* = 0.176) (Supplementary Table S7). Only 67 patients (11.9%) had hs-cTnT above 28.42 ng/L, of whom 41 had a poor 90-day outcome, and 52 patients (9.2%) had hs-cTnT above 31.68 ng/L, among whom 27 deaths occurred; the attenuation of the gradient above the turning points is therefore estimated from sparse data. These values should be interpreted as model-derived turning points within this dataset rather than validated clinical cutoffs.

**Figure 4:**
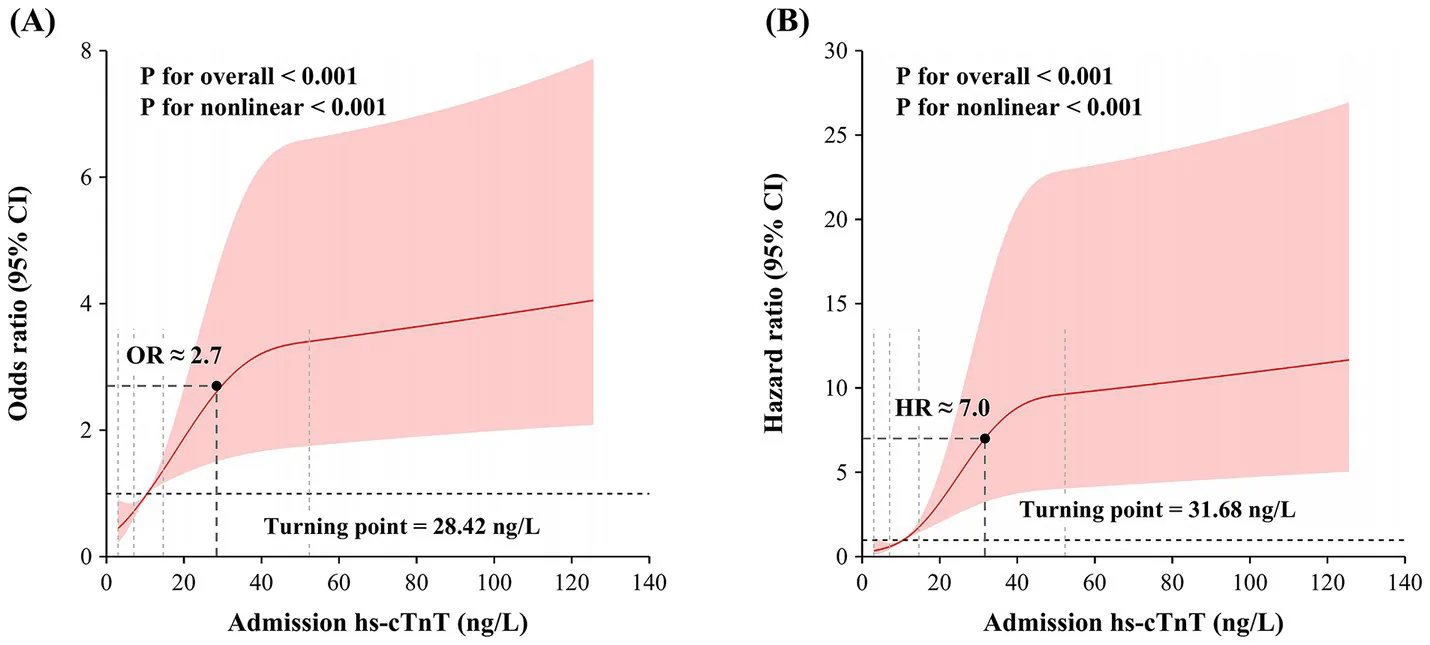
Restricted cubic spline curves showing the association between hs-cTnT and outcomes. **(A)** Association between hs-cTnT and the risk of poor functional outcome at 90 days (adjusted Model 3). The red solid line represents odds ratios with 95% confidence intervals (shaded area). **(B)** Association between hs-cTnT and the risk of mortality (adjusted Model 3). The red solid line represents hazard ratios with 95% confidence intervals (shaded area). For both panels, P for overall and P for nonlinearity were < 0.001. The vertical and horizontal dashed lines indicate the model-derived turning point at 28.42 ng/L **(A)** / 31.68 ng/L **(B)**, estimated from segmented regression. These values should be interpreted as exploratory rather than validated clinical cutoffs.

## 4 Discussion

### 4.1 Main findings

This study systematically investigated the association of admission hs-cTnT with 90-day functional outcome and all-cause mortality during follow-up in patients with acute ischemic stroke, with particular attention to potential nonlinear patterns. Three findings merit emphasis. Admission hs-cTnT >14 ng/L was independently associated with worse 90-day function and higher mortality; these associations were nonlinear; and an exploratory model-derived inflection was observed at approximately 28–32 ng/L. Overall, these findings support a role for hs-cTnT as an adjunctive marker of prognosis rather than as a standalone threshold for treatment decisions.

### 4.2 Comparison with previous studies

Our findings are consistent with and extend previous research on the prognostic value of hs-cTnT in ischemic stroke. Multiple studies have shown that elevated cardiac troponin levels are common in patients with acute ischemic stroke and are associated with adverse outcomes (22, 23). A meta-analysis including 12 studies and more than 7,900 patients confirmed that troponin elevation is an independent predictor of mortality after acute stroke (24). Similarly, a cohort study involving 516 patients showed that elevated hs-cTnT was associated with increased mortality risk, with each standard deviation increase corresponding to a hazard ratio of 3.14 for all-cause mortality (25).

However, most previous studies have treated hs-cTnT as either a binary variable (elevated vs. normal) or assumed a linear relationship with outcomes, potentially oversimplifying the true nature of this association. Our study adds to the existing literature by using restricted cubic spline regression to characterize the nonlinear dose–response relationship, suggesting that the risk increment was attenuated beyond certain concentrations. Similar nonlinear associations between hs-cTn and clinical events have also been reported in broader cardiovascular populations (26). Nevertheless, the apparent attenuation of risk beyond the turning point should be interpreted cautiously, because the turning points were model-derived within a single cohort, have not been externally validated, and the statistical power above the turning point was limited.

Notably, our study found that elevated hs-cTnT was more strongly associated with mortality than with functional outcomes. This differential prognostic value may reflect the multifactorial nature of post-stroke functional recovery, which is influenced by factors beyond cardiac status, including stroke location, rehabilitation intensity, and social support. In contrast, mortality may be more directly linked to cardiovascular complications and overall physiologic reserve, both of which are reflected by hs-cTnT levels. A comparable pattern has been reported in a prospective cohort of patients with first-ever ischemic stroke, in which hs-cTnT was more strongly associated with death and with major cardiovascular events than with recurrent stroke (27).

### 4.3 Potential mechanisms underlying hs-cTnT elevation and stroke prognosis

The mechanisms underlying elevated hs-cTnT in acute ischemic stroke are complex and reflect a bidirectional cardio-cerebral interaction. One proposed mechanism is neurogenic cardiac injury, in which acute brain ischemia, particularly when involving the insular cortex or autonomic centers, may trigger a systemic catecholamine surge. This sympathetic hyperactivation has been hypothesized to cause direct cardiomyocyte toxicity and myocardial stunning (for example, Takotsubo-like cardiomyopathy), with troponin release occurring even in the absence of overt coronary occlusion (23, 28). However, catecholamine concentrations and serial echocardiography were not obtained, so this mechanism remains speculative in our cohort.

Beyond acute neurogenic stress, admission hs-cTnT may also reflect the burden of underlying cardiovascular comorbidities. In our cohort, elevated hs-cTnT was associated with older age, hypertension, and coronary heart disease, consistent with the contribution of shared vascular risk factors and subclinical myocardial injury (29). However, these associations are observational and do not establish causality. In addition, stroke severity may be relevant. In our cohort, patients with elevated hs-cTnT had significantly higher NIHSS scores. This observation is consistent with the hypothesis that more severe stroke may provoke greater autonomic dysregulation and more pronounced cardiac injury, although our data cannot establish a causal pathway. This interaction may further worsen both neurologic and cardiac injury through hemodynamic instability and reduced physiologic reserve.

Systemic inflammation has also been proposed as a potential mediator. Acute ischemic stroke is known to trigger an inflammatory cascade that may contribute to atherosclerotic plaque destabilization and myocardial cell injury (30); however, inflammatory biomarkers were not measured in this study, and this mechanism remains hypothetical. Finally, reduced renal clearance may contribute to higher hs-cTnT levels. In our cohort, patients with elevated hs-cTnT had significantly lower eGFR, consistent with the known effect of renal function on troponin clearance. The prognostic association nonetheless persisted after adjustment for eGFR, so renal function alone does not fully explain the relationship; hs-cTnT more likely reflects a composite risk profile that includes, but is not limited to, cardiovascular burden and systemic vulnerability. The specific contributions of neurogenic stress, inflammation, and renal clearance cannot be quantified in our dataset and warrant further study.

### 4.4 Clinical implications for prognostic assessment and neuro-cardiac evaluation

From a clinical perspective, the main value of admission hs-cTnT in AIS may lie in prognostic assessment rather than in defining an immediate threshold-based management strategy. The model-derived turning points observed in our study should therefore be interpreted as exploratory signals of risk transition rather than as definitive bedside cutoffs. Elevated hs-cTnT may help identify patients with a higher burden of cardiovascular comorbidity or systemic stress; however, our study did not specifically diagnose covert cardiac injury or stroke-heart syndrome. Importantly, because this was an observational study without a randomized or interventional design, our findings cannot establish treatment recommendations. In particular, we cannot conclude that serial troponin testing, electrocardiographic monitoring, echocardiography, or cardiology referral improves outcomes. These strategies may be reasonable to explore in future prospective studies, but they should not be presented as recommendations derived from the present analysis. The nonlinear pattern observed in our study supports the view that hs-cTnT contains more prognostic information than a simple normal/abnormal dichotomy, but it does not indicate that a specific concentration should directly trigger a fixed treatment algorithm.

### 4.5 Strengths and limitations

Our study has several strengths. We used restricted cubic spline regression to characterize the nonlinear relationship between hs-cTnT and clinical outcomes, thereby providing a more nuanced assessment than conventional linear or categorical analyses. We also systematically investigated both short-term functional outcomes and all-cause mortality during follow-up. In addition, exploratory segmented analyses were performed to estimate model-derived turning points, which may help refine continuous prognostic assessment.

However, several limitations should be acknowledged. First, 49 of the 613 clinically eligible patients (8.0%) were excluded because data required for the prespecified analyses were incomplete, including without an hs-cTnT result within 24 h of admission; although this proportion is small, complete-case analysis may still have introduced selection bias of uncertain direction. Second, patients who died before sampling could not be included, and the results therefore apply to patients who survived the first hours after admission. We used admission as the time origin so that the interval before exposure ascertainment was not counted as time at risk, and onset-based specifications gave similar estimates (Supplementary Table S5); nevertheless, this design cannot entirely exclude survivor bias in the earliest phase after stroke. Third, despite adjustment for multiple clinical confounders, residual confounding remains possible, particularly because we lacked comprehensive echocardiographic data, serial electrocardiographic changes, and other complementary cardiac biomarkers. Fourth, this was a single-center retrospective study conducted in a county hospital in China, which may limit generalizability to other settings and populations. Fifth, hs-cTnT was measured only once on admission, and the absence of serial troponin data prevented assessment of dynamic troponin trajectories. Sixth, the exploratory turning points (28–32 ng/L) were model-derived within this dataset and have not been prospectively or externally validated; they should therefore be interpreted as exploratory signals rather than as definitive clinical cutoffs. Finally, the median follow-up of 17 months was relatively short, detailed data on specific causes of death were unavailable, and because this was an observational study, our findings cannot establish treatment recommendations. Future prospective multicenter studies with systematic serial hs-cTnT measurement and external validation are needed to confirm our findings.

## 5 Conclusion

In conclusion, in this single-center retrospective cohort of patients with acute ischemic stroke, admission hs-cTnT was independently associated with 90-day functional outcome and all-cause mortality during follow-up. The association appeared to be nonlinear across the biomarker range, with exploratory model-derived turning points at approximately 28–32 ng/L. However, these values were derived from a single cohort, have not been externally validated, and should not be used to guide clinical decision-making. Prospective, multicenter studies with external validation are required before the proposed 28–32 ng/L values can be considered for any clinical application. At present, the main clinical relevance of hs-cTnT may lie in complementing early prognostic assessment, but this observational study cannot establish treatment recommendations.

## Data Availability

The original contributions presented in the study are included in the article/Supplementary material, further inquiries can be directed to the corresponding author.

## Supplementary material

SUPPLEMENTARY FIGURE S1 Subgroup analyses of the associations between hs-cTnT and adverse outcomes. **(A)** Subgroup analyses for 90-day poor functional outcome (mRS ≥3), presented as odds ratios (ORs) per 1 ng/L increase in hs-cTnT. **(B)** Subgroup analyses for long-term all-cause mortality, presented as hazard ratios (HRs) per 1 ng/L increase in hs-cTnT. Estimates were derived from fully adjusted models (Model 3). The solid squares indicate effect estimates with 95% confidence intervals, and the dashed line represents the null value. DM, diabetes mellitus; CHD, coronary heart disease.
