# Supplementary material for: Admission high-sensitivity cardiac troponin T and clinical outcomes after acute ischemic stroke: a retrospective cohort study with exploratory nonlinear analysis
Source: Front Neurol. 2026 Oct 22;17:1882492. doi: 10.3389/fneur.2026.1882492 (PMC13735727; doi:10.3389/fneur.2026.1882492)
Supplement: Supplementary file 1 [file Table_1.docx]

**Table S1.** Association between hs-cTnT quartiles and all-cause mortality in Cox proportional hazards models

| Admission hs-cTnT quartile (ng/L) | Model 1  HR (95% CI) | P value | Model 2  HR (95% CI) | P value | Model 3  HR (95% CI) | P value |
| --- | --- | --- | --- | --- | --- | --- |
| <5.10 | 1.00 (Reference) |  | 1.00 (Reference) |  | 1.00 (Reference) |  |
| 5.10–10.54 | 3.88 (1.07–13.79) | 0.039 | 3.70 (1.03–13.30) | 0.045 | 3.39 (0.94–12.28) | 0.063 |
| 10.55–17.09 | 5.77 (1.68–19.82) | 0.005 | 5.70 (1.64–19.84) | 0.006 | 4.79 (1.36–16.80) | 0.015 |
| >17.09 | 19.13 (5.96–61.42) | <0.001 | 17.35 (5.33–56.56) | <0.001 | 10.66 (3.12–36.50) | <0.001 |
| P for trend | <0.001 |  | <0.001 |  | <0.001 |  |

*Note. HR, hazard ratio; CI, confidence interval; hs-cTnT, high-sensitivity cardiac troponin T. Model 1 was unadjusted. Model 2 was adjusted for age, sex, and smoking. Model 3 was additionally adjusted for baseline NIHSS score, hypertension, diabetes mellitus, coronary heart disease, prior cerebral infarction, history of renal impairment, and eGFR.*
