# Supplementary material for: Admission high-sensitivity cardiac troponin T and clinical outcomes after acute ischemic stroke: a retrospective cohort study with exploratory nonlinear analysis
Source: Front Neurol. 2026 Oct 22;17:1882492. doi: 10.3389/fneur.2026.1882492 (PMC13735727; doi:10.3389/fneur.2026.1882492)
Supplement: Supplementary file 2 [file Table_2.docx]

**Table S2.** Multivariable associations of continuous hs-cTnT with 90-day poor functional outcome and long-term all-cause mortality

| Outcome | hs-cTnT increment | Model 1 OR / HR (95% CI) | P value | Model 2 OR / HR (95% CI) | P value | Model 3  OR / HR (95% CI) | P value |
| --- | --- | --- | --- | --- | --- | --- | --- |
| 90-day poor functional outcome (mRS 3–6) | Per 1 ng/L | 1.012 (1.004 ~ 1.021) | 0.004 | 1.010 (1.003 ~ 1.017) | 0.004 | 1.005 (1.001 ~ 1.010) | 0.028 |
| Long-term all-cause mortality | Per 1 ng/L | 1.001 (1.001 ~ 1.001) | <0.001 | 1.001 (1.001 ~ 1.001) | <0.001 | 1.001 (1.001 ~ 1.001) | 0.004 |
| 90-day poor functional outcome (mRS 3–6) | Per 10 ng/L | 1.130 (1.040 ~ 1.228) | 0.004 | 1.109 (1.034 ~ 1.189) | 0.004 | 1.053 (1.006 ~ 1.101) | 0.028 |
| Long-term all-cause mortality | Per 10 ng/L | 1.008 (1.004 ~ 1.013) | <0.001 | 1.007 (1.003 ~ 1.012) | <0.001 | 1.007 (1.002 ~ 1.011) | 0.004 |

OR, odds ratio; HR, hazard ratio; CI, confidence interval; hs-cTnT, high-sensitivity cardiac troponin T; mRS, modified Rankin Scale. For 90-day poor functional outcome, multivariable logistic regression was used and estimates are ORs; for long-term all-cause mortality, Cox proportional hazards models were used and estimates are HRs. Model 1 was unadjusted. Model 2 was adjusted for age, sex, and smoking status. Model 3 was further adjusted for baseline NIHSS score, hypertension, diabetes mellitus, coronary heart disease, prior cerebral infarction, history of renal impairment, and eGFR. The per 10 ng/L estimates were obtained by rescaling the continuous hs-cTnT variable and refitting the corresponding regression models using the same covariates and analytic population.
