# Supplementary material for: Admission high-sensitivity cardiac troponin T and clinical outcomes after acute ischemic stroke: a retrospective cohort study with exploratory nonlinear analysis
Source: Front Neurol. 2026 Oct 22;17:1882492. doi: 10.3389/fneur.2026.1882492 (PMC13735727; doi:10.3389/fneur.2026.1882492)
Supplement: Supplementary file 3 [file Table_3.docx]

## Table S3. Sensitivity analysis using a parsimonious Cox proportional hazards model for long-term all-cause mortality

| Variable | Univariate HR (95% CI) | P | Parsimonious multivariable HR (95% CI) | P |
| --- | --- | --- | --- | --- |
| hs-cTnT >14 ng/L | 6.24 (3.68–10.57) | <0.001 | 3.59 (1.98–6.50) | <0.001 |
| Age, per year | 1.07 (1.04–1.10) | <0.001 | 1.03 (1.01–1.06) | 0.031 |
| Male sex | 1.87 (1.20–2.92) | 0.006 | 1.15 (0.70–1.87) | 0.582 |
| NIHSS, per point | 1.15 (1.12–1.18) | <0.001 | 1.11 (1.07–1.14) | <0.001 |
| eGFR, per mL/min/1.73 m² | 0.98 (0.97–0.99) | 0.002 | 1.00 (0.99–1.01) | 0.868 |

*The parsimonious multivariable Cox model included admission hs-cTnT (>14 vs ≤14 ng/L), age, sex, admission NIHSS, and eGFR. This sensitivity analysis was performed to reduce model complexity given the relatively limited number of all-cause mortality events.*
