# Supplementary material for: Admission high-sensitivity cardiac troponin T and clinical outcomes after acute ischemic stroke: a retrospective cohort study with exploratory nonlinear analysis
Source: Front Neurol. 2026 Oct 22;17:1882492. doi: 10.3389/fneur.2026.1882492 (PMC13735727; doi:10.3389/fneur.2026.1882492)
Supplement: Supplementary file 4 [file Table_4.docx]

## Table S4. Assessment of the proportional hazards assumption for the multivariable Cox regression model

| Covariate | χ² | df | P value |
| --- | --- | --- | --- |
| hs-cTnT (>14 vs ≤14 ng/L) | 3.050 | 1 | 0.081 |
| Age | 0.003 | 1 | 0.959 |
| Sex | 0.013 | 1 | 0.909 |
| Smoking | 1.447 | 1 | 0.229 |
| NIHSS score at admission | 0.134 | 1 | 0.715 |
| Hypertension | 1.733 | 1 | 0.188 |
| Diabetes mellitus | 0.904 | 1 | 0.342 |
| Coronary heart disease | 0.422 | 1 | 0.516 |
| Previous cerebral infarction | 0.518 | 1 | 0.472 |
| History of renal impairment | 0.000 | 1 | 1.000 |
| eGFR | 2.103 | 1 | 0.147 |
| Global test | 12.036 | 11 | 0.361 |

The proportional hazards assumption was assessed using Schoenfeld residual-based tests. A P value < 0.05 was considered indicative of potential violation. No statistically significant evidence of violation was observed for any individual covariate or for the global test, although the test statistic for hs-cTnT approached conventional significance (P = 0.081). History of renal impairment was present in only 9 patients (1.6%), so the test statistic for this covariate is uninformative. Variable renamed from “Kidney disease” to “History of renal impairment” for consistency with the Methods, Table 1 and Table 2.
