# Supplementary material for: Admission high-sensitivity cardiac troponin T and clinical outcomes after acute ischemic stroke: a retrospective cohort study with exploratory nonlinear analysis
Source: Front Neurol. 2026 Oct 22;17:1882492. doi: 10.3389/fneur.2026.1882492 (PMC13735727; doi:10.3389/fneur.2026.1882492)
Supplement: Supplementary file 5 [file Table_5.docx]

### **Table S5.** Sensitivity analysis of the association between admission hs-cTnT and long-term all-cause mortality under different time-origin definitions

| Time-origin definition | N | Deaths | Fully adjusted HR (95% CI) | P value |
| --- | --- | --- | --- | --- |
| Hospital admission | 564 | 78 | 4.12 (2.31–7.48) | <0.001 |
| Onset To Admission | 564 | 78 | 4.17 (2.33–7.46) | <0.001 |
| Admission To Sampling | 564 | 78 | 4.41 (2.46–7.88) | <0.001 |

Note：The primary analysis defined hospital admission as the time origin. In the first sensitivity analysis, stroke onset was defined as the time origin without delayed entry. In the second sensitivity analysis, stroke onset was retained as the time origin, but patients entered the risk set at hospital admission using delayed entry. All sensitivity analyses used the same prespecified multivariable Cox model as the primary analysis, adjusting for age, sex, smoking status, admission NIHSS score, hypertension, diabetes mellitus, coronary heart disease, prior cerebral infarction, history of renal impairment, and eGFR

*.*
