# Supplementary material for: Admission high-sensitivity cardiac troponin T and clinical outcomes after acute ischemic stroke: a retrospective cohort study with exploratory nonlinear analysis
Source: Front Neurol. 2026 Oct 22;17:1882492. doi: 10.3389/fneur.2026.1882492 (PMC13735727; doi:10.3389/fneur.2026.1882492)
Supplement: Supplementary file 6 [file Table_6.docx]

Table S6. Fine–Gray competing risk regression analysis for recurrent cerebrovascular events considering death as a competing event

| Variables | Unadjusted SHR (95% CI) | P value | Adjusted SHR (95% CI) | P value |
| --- | --- | --- | --- | --- |
| hs-cTnT >14 ng/L | 0.87 (0.50–1.51) | 0.618 | 1.05 (0.56–1.96) | 0.877 |
| Age, per year | — | — | 0.99 (0.96–1.01) | 0.403 |
| Male sex | — | — | 1.32 (0.73–2.37) | 0.355 |
| NIHSS score at admission, per point | — | — | 0.97 (0.91–1.04) | 0.449 |
| eGFR, per mL/min/1.73 m² | — | — | 1.00 (0.99–1.01) | 0.921 |
| Coronary heart disease | — | — | 1.23 (0.57–2.68) | 0.599 |

SHR, subdistribution hazard ratio; CI, confidence interval; hs-cTnT, high-sensitivity cardiac troponin T; NIHSS, National Institutes of Health Stroke Scale; eGFR, estimated glomerular filtration rate. Death without a prior recurrent cerebrovascular event was treated as a competing event. Cumulative incidence functions were compared between hs-cTnT groups using Gray's test (P = **0.695**). The adjusted model was adjusted for age, sex, admission NIHSS score, eGFR, and coronary heart disease; a reduced covariate set was used, rather than the full Model 3 covariates, because only 71 recurrent events were observed.
