# Supplementary material for: Admission high-sensitivity cardiac troponin T and clinical outcomes after acute ischemic stroke: a retrospective cohort study with exploratory nonlinear analysis
Source: Front Neurol. 2026 Oct 22;17:1882492. doi: 10.3389/fneur.2026.1882492 (PMC13735727; doi:10.3389/fneur.2026.1882492)
Supplement: Supplementary file 7 [file Table_7.docx]

Table S7. Exploratory turning-point analysis of hs-cTnT for 90-day poor functional outcome and all-cause mortality

| Outcome | Model | Turning point (ng/L) | Effect (95% CI) | P value | P for likelihood ratio test |
| --- | --- | --- | --- | --- | --- |
| 90-day poor functional outcome | Single-slope logistic model | — | 1.004 (1.000–1.008) | 0.012 | — |
|  | Two-piecewise logistic model | 28.42 | <28.420 ng/L:1.048 (1.005–1.093) | <0.001 | <0.001 |
|  |  |  | ≥28.420 ng/L: 1.002 (0.997–1.006) | 0.328 |  |
| All-cause mortality | Single-slope Cox model | — | 1.007 (1.002–1.011) | 0.002 | — |
|  | Two-piecewise Cox model | 31.68 | <31.680 ng/L: 1.096 (1.045–1.149) | <0.001 | <0.001 |
|  |  |  | ≥31.680 ng/L: 1.005 (0.999–1.012) | 0.176 |  |

All effect estimates are expressed per 1 ng/L increase in hs-cTnT. Effect sizes are odds ratios (ORs) for 90-day poor functional outcome and hazard ratios (HRs) for all-cause mortality, with 95% confidence intervals (CIs). Turning points were estimated using segmented regression models and are exploratory, model-derived values within this dataset rather than validated clinical cutoffs. P for likelihood ratio test compares the fit of the two-piecewise model with the corresponding single-slope model.
